# Scrotal cutaneous verruciform xanthoma with monocyte chemoattractant protein-1 immunohistochemical study: a case report

**DOI:** 10.1186/1752-1947-6-260

**Published:** 2012-08-31

**Authors:** Chihiro Ito, Riko Kitazawac, Kenji Makita, Takafumi Watanabe, Akihiro Toda, Ryuma Haraguchi, Shinji Tanaka, Sohei Kitazawa

**Affiliations:** 1 Division of Molecular Pathology, Ehime University Graduate School of Medicine, Shitsukawa, Toon City, Ehime, 791-0295, Japan; 2 Department of Plastic Surgery, Ishikawa Hospital, Kamibun-cho 732-1, Shikoku-chuo City, Ehime, 799-0121, Japan

## Abstract

**Introduction:**

Verruciform xanthoma is a rare, benign lesion characterized by hyperkeratosis and aggregates of foam cell macrophages. Here, we describe a case of verruciform xanthoma on the scrotum, in which the immunohistochemical localization of monocyte chemoattractant protein-1, a chemokine of the C-C or beta family that has been shown to induce the recruitment of monocytes for injured tissue, was analyzed to determine which cells release chemoattractants for macrophages.

**Case presentation:**

A 75-year-old Japanese man with a well-defined nodule on the left scrotum was admitted to the hospital. An excision biopsy revealed epidermal papillary proliferation with parakeratosis, hyperkeratosis, and infiltration of foam cell macrophages, whereby a pathological diagnosis of benign cutaneous verruciform xanthoma was made. Immunohistochemically, monocyte chemoattractant protein-1 was observed predominantly on cytokeratin AE1/AE3-positive differentiating keratinocytes in the prickle cell layer. However, while infiltrating macrophages were densely stained for monocyte chemoattractant protein-1, keratinocytes in the basal and parabasal layers were almost negative.

**Conclusions:**

We demonstrated that keratinocyte-derived monocyte chemoattractant protein-1 plays an important role in the establishment of particular histological features of verruciform xanthoma. However, in the present case, unlike in previous reports, monocyte chemoattractant protein-1 immunostaining in keratinocytes in the basal and parabasal layers was not prominent. We speculate that in the active phase of verruciform xanthoma, when continuous stimuli that release monocyte chemoattractant protein-1 from keratinocytes to the surrounding stromal area are present, the apparent immunostaining of monocyte chemoattractant protein-1 can be underestimated because of the void created by accelerated keratinocyte release from the cytoplasmic fraction.

## Introduction

Verruciform xanthoma (VX) is an uncommon, benign lesion first reported in the oral cavity in 1971 [1]. A survey of 282 cases of VX involving different mucocutaneous sites has established the lesion as a distinct clinicopathologic entity [2]. It occurs mainly in oral mucosa and occasionally at extra-oral sites, including those on the penis [3], the scrotum [4], and the vulva [5]. Clinically, the lesion is painless, asymptomatic, slow growing (up to 2cm in size), and slightly elevated with a yellowish, reddish, or grayish rough and granular surface [6], mimicking conventional papilloma, verrucous carcinoma and squamous cell carcinoma [7]. Histologically, VX is characterized by papillomatosis, parakeratosis, and accumulation of foam cell macrophages [1,8,9].

This study describes a case of VX on the scrotum and analyzes the immunohistochemical localization of the major macrophage chemotactic factor, monocyte chemoattractant protein-1 (MCP-1), to elucidate the particular tumor-macrophage interaction in VX.

## Case presentation

**Figure 1:**
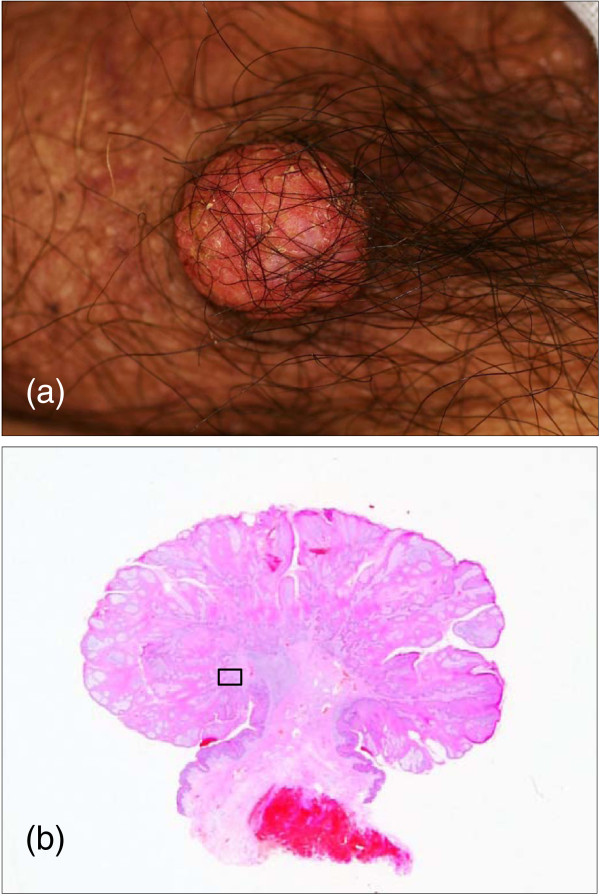
**(a) Pedunculated, rubbery soft, red nodule (13mm in diameter) with a mulberry-like surface on the scrotum. (b)** The lesion consists of pseudocarcinomatous acanthotic epidermis and stroma with dilated vessels under low magnification (hematoxylin and eosin stain). High magnification of the boxed area in Figure 2(a).

**Figure 2:**
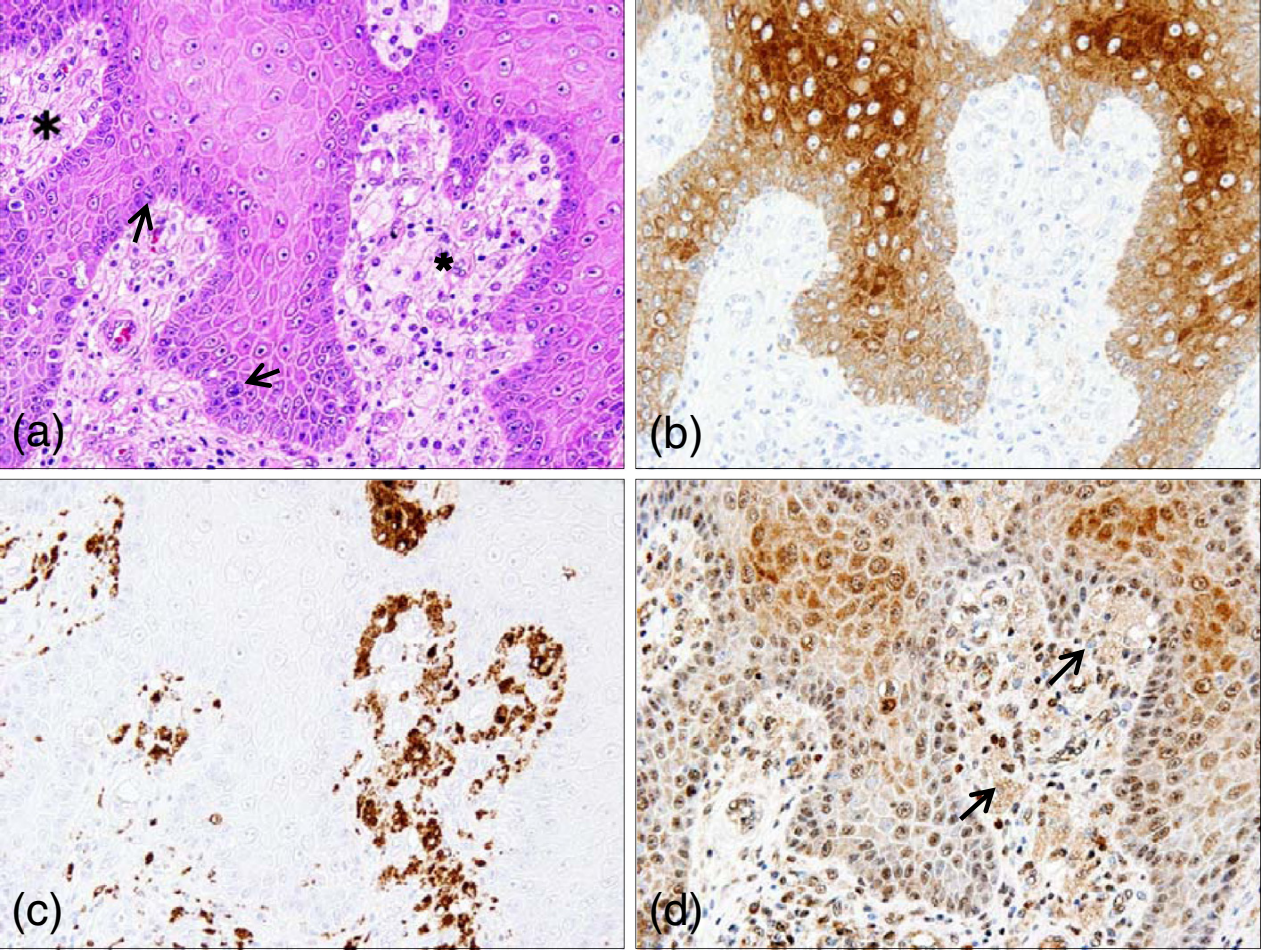
**Immunohistochemical evaluation of chemoattractants for macrophages. (a)** Hematoxylin and eosin staining shows verruciform xanthoma consisting of hyperkeratosis, a few mitotic figures (arrows) and infiltrating foamy cells (asterisks, ×200). **(b)** Cytokeratin AE1/AE3 showing positive immunostaining in the epithelial cells of the verruciform xanthoma (×200). **(c)** CD68 showing positive cytoplasmic immunostaining exclusively in the majority of foam cells infiltrating beneath the basal cells (×200). **(d)** The parts densely stained for monocyte chemoattractant protein-1 (MCP-1) are observed in the differentiating keratinocytes over the basal and parabasal layers. Clusters of the infiltrating macrophages are also stained positive, albeit less intensely, for MCP-1 (arrows, ×200).

A 75-year-old Japanese man was admitted to our hospital with a gradually growing cutaneous polypoid mass that had appeared on the skin of the left scrotum approximately one year before. The tumor measured 13mm in diameter with relatively well-defined whitish-yellow outlines (Figure 1a). Histological examination of an excisional biopsy revealed epidermal papillary proliferation with parakeratosis, hyperkeratosis, and neutrophil infiltration (Figures 1b and 2a). Although mitotic figures were noted in the basal layer (Figure 2a, arrows), cellular atypia was not prominent. Besides abundant plasma cells in the upper dermis, numerous foamy macrophages infiltrated the dermal papillae, forming a characteristic clear zone beneath the basement membrane (Figure 2a, asterisks). Histopathologically, the tumor was diagnosed as a benign cutaneous verruciform xanthoma with negative lateral and deep surgical margins.

To determine which cells release chemoattractants for macrophages, formalin-fixed and paraffin-embedded sections were stained for cytokeratin (AE1/AE3, M3515; Dako, Carpinteria, CA, USA; 1:200), CD68 (M0876; Dako; 1:100) and MCP-1 (DA103; BD Biosciences, San Diego, CA, USA; 1:40, 1:200, 1:800). After microwave heat-induced epitope retrieval, endogenous peroxidase activity was blocked with hydrogen peroxide (H_2_O_2_) in methanol. Indirect immunohistochemistry with the use of horseradish peroxidase conjugated anti-mouse rabbit antibody revealed that cytokeratin AE1/AE3 was strongly positive in differentiating epidermal keratinocytes, and weakly positive in keratinocytes in the basal and parabasal layers (Figure 2b). However, strong CD68 staining was observed almost exclusively in foamy cells infiltrating beneath the basal cells (Figure 2c). The parts densely stained for MCP-1 were observed in the differentiating cytokeratin AE1/AE3-positive keratinocytes. Clusters of the infiltrating macrophages also stained positive for MCP-1 (Figure 2d).

## Discussion

Previous studies revealed a possible pathogenesis of VX that included the local release of lipid by damaged keratinocytes through inflammation [10]. Under the assumption that MCP-1 produced by the proliferating keratinocytes, especially by those with close contact with foamy macrophages, plays some roles in developing this particular histological feature, we investigated the immunohistochemical localization of MCP-1. In the present case, however, MCP-1 expression was observed in keratinocytes in the papilloma lesion, but its localization was observed predominantly in differentiating keratinocytes in the prickle cell layer.

MCP-1, produced by many types of cells, is a chemokine of the C-C or beta family that has been shown to induce the recruitment of monocytes for injured tissue; its excessive production by keratinocytes has been implicated in psoriasis and other inflammatory skin diseases; transgenic mice that express murine MCP-1 in the basal layer of epidermis do not, however, exhibit spontaneous cutaneous inflammation or any other discernible skin pathology, but show hypersensitivity responses to elicited inflammation in the skin by the recruitment of dendritic and Langerhans cells [11]. One of the apparent pathophysiological roles of MCP-1 in the skin is, therefore, chemotaxis of immunomodulators to the skin, and the overexpression of MCP-1 *per se* may be a requisite but not sufficient condition for causing VX.

A previous immunohistochemical study on a series of VX cases has revealed that MCP-1 localizes in the basal layer of the epidermis [12]. To explain an aspect of such immunohistochemical differences, we formulated two hypotheses: firstly, scarring beneath the basal layer prevented basal cells from releasing MCP-1, as reported for keloid-derived fibroblasts [13], and secondly, accelerated release of MCP-1 exhausted significant amounts of MCP-1 from the cytoplasm of keratinocytes in the basal and parabasal layers. It is well known that the epidermis shows a reparative phenotype when overlying a scar or the sclerotic dermis of lichen sclerosus [13,14]. This stromal-keratinocyte interaction is believed to account for the change of keratin AE1 expression from from basal keratinocytes in normal skin to spinous keratinocytes in scars and lichen sclerosus. Thus, our finding of MCP-1 expression in spinous keratinocytes, rather than the basal layer, may be the consequence of an altered dermal/stromal phenotype. It is likely not coincidence that both lichen sclerosus and verruciform xanthoma also show dermal lymphedema; the latter is thought to be the consequence of the former [15]. Additionally, when continuous stimuli to release MCP-1 from keratinocytes to the surrounding stromal area is present, apparent immunostaining of MCP-1 can be underestimated. Also, MCP-1 immunohistochemically localizes in infiltrating or aggregating macrophages themselves [16]. Thus, macrophages recruited by MCP-1 may sustain themselves in both paracrine and autocrine ways.

## Conclusions

Although other factors associated with MCP-1 that characterize particular histopathological features of VX have not yet been established, we speculate that MCP-1 expression in proliferating and differentiating keratinocytes may have a supplemental role in the establishment of VX.

## Consent

Written informed consent was obtained from the patient for publication of this case report and any accompanying images. A copy of the written consent is available for review by the Editor-in-Chief of this journal.

## Competing interests

The authors declare that they have no competing interests.

## Authors’ contributions

TI and SK were involved in the whole process. RK, ST and AT analyzed the clinical case. KM and TW assisted with clinical aspects. RH was involved in hematoxylin and eosin staining and immunostaining. All authors read and approved the final version of the manuscript.
